# Wnt9A Induction Linked to Suppression of Human Colorectal Cancer Cell Proliferation

**DOI:** 10.3390/ijms17040495

**Published:** 2016-04-02

**Authors:** Irshad Ali, Bani Medegan, Donald P. Braun

**Affiliations:** 1 Senior Research Associate, Translational Research Laboratory, Cancer Treatment Centers of America^®^, 2520 Elisha Avenue, Zion, IL 60099, USA; 2 Research Associate, Translational Research Laboratory, Cancer Treatment Centers of America^®^, 2520 Elisha Avenue, Zion, IL 60099, USA; Bani.Fagla@ctca-hope.com; 3 VP Translational Research and Chief Science Officer, Cancer Treatment Centers of America^®^, 2610 Sheridan Rd., Zion, IL 60099, USA; Donald.Braun@ctca-hope.com

**Keywords:** Wnt9A, β-catenin, non-canonical Wnt pathway, canonical Wnt pathway, Wnt pathway ligand, LiCl, human colorectal cancer

## Abstract

Most studies of Wnt signaling in malignant tissues have focused on the canonical Wnt pathway (CWP) due to its role in stimulating cellular proliferation. The role of the non-canonical Wnt pathway (NCWP) in tissues with dysregulated Wnt signaling is not fully understood. Understanding NCWP’s role is important since these opposing pathways act in concert to maintain homeostasis in healthy tissues. Our preliminary studies demonstrated that LiCl inhibited proliferation of primary cells derived from colorectal cancer (CRC). Since LiCl stimulates cell proliferation in normal tissues and NCWP suppresses it, the present study was designed to investigate the impact of NCWP components in LiCl-mediated effects. LiCl-mediated inhibition of CRC cell proliferation (*p* < 0.001) and increased apoptosis (*p* < 0.01) coincided with 23-fold increase (*p* < 0.025) in the expression of the NCWP ligand, *Wnt9A*. LiCl also suppressed β-catenin mRNA (*p* < 0.03), total β-catenin protein (*p* < 0.025) and the active form of β-catenin. LiCl-mediated inhibition of CRC cell proliferation was partially reversed by IWP-2, and *Wnt9A* antibody. Recombinant *Wnt9A* protein emulated LiCl effects by suppressing β-catenin protein (*p* < 0.001), inhibiting proliferation (*p* < 0.001) and increasing apoptosis (*p* < 0.03). This is the first study to demonstrate induction of a NCWP ligand, *Wnt9A* as part of a mechanism for LiCl-mediated suppression of CRC cell proliferation.

## 1. Introduction

Wnt signaling is an intricate ensemble of components of both canonical and non-canonical pathways involved with various processes including cellular proliferation, differentiation, motility and polarity [1]. Although several components are shared between the canonical and non-canonical pathways, the principal point of divergence entails the utilization of β-catenin. In the off state, in the absence of Wnt ligands, β-catenin, a second messenger utilized by the canonical pathway, is produced in a constitutive manner but is degraded by the action of the destruction complex. Activation of the canonical pathway leads to inhibition of the destruction complex resulting in accumulation of β-catenin in the cytosol followed by its translocation into the nucleus. In the nucleus, β-catenin binds to transcription factors, T-cell factor/lymphocyte enhancer factor (TCF/LEF) and turns on genes relevant for cell proliferation. The non-canonical Wnt pathway does not utilize β-catenin but instead, uses various other components for its activities [1]. To prevent uncontrolled cell proliferation, the canonical pathway is stringently regulated by various inhibitors, modulators and stimulators [2].

An added layer of regulation is provided by the non-canonical pathway which also tempers canonical pathway activity [3]. Thus, activation of the non-canonical Wnt pathway stimulates cellular differentiation and opposes cellular proliferation mediated by the canonical Wnt pathway. In healthy tissues these opposing pathways act in concert to maintain homeostasis [4,5]. One of the characteristic features of malignant tissues is disturbance of Wnt pathway signaling wherein components of the canonical and non-canonical pathways are modulated in a disproportionate manner leading to dysfunction of normal homeostatic mechanisms in cancer tissues.

Most studies of Wnt signaling in malignant tissues have focused on the canonical pathway due to its role in stimulating cellular proliferation. These studies have been instrumental in demonstrating that a prominent feature of Wnt signaling dysfunction in cancer cells involves the regulation of β-catenin. Due to mutations in either the gene(s) for β-catenin or the destruction complex, the protein is relieved from regulatory constraints and is able to stimulate target gene expression in an uncontrolled manner [6]. It is important to appreciate, however, that mechanisms other than the dysregulation of β-catenin have been shown to contribute to uncontrolled proliferation in cancer [7].

Given the importance of the non-canonical pathway in normal tissue homeostasis, it is conceivable that components of the non-canonical pathway might also contribute to the abnormal proliferation of malignant tissues. Demonstration that non-canonical components are suppressed in some cancers [8,9,10] is consistent with this hypothesis. These considerations provide a rationale for studying the function of the non-canonical Wnt pathway in malignant cells and the role this may play in the development and progression of different human cancers.

Recent studies show that lithium chloride, a drug used for psychotropic disorders [11], suppresses cancer cell proliferation [12,13]. The inhibition by LiCl is contradictory to the effect observed in normal tissues. In normal tissues LiCl-mediated inhibition of GSK3β leads to stabilization of β-catenin and activation of the canonical Wnt pathway that stimulates cell proliferation [14]. In cancer tissues however LiCl has been shown to both inhibit [12] and stimulate [15] cell proliferation which suggests that mechanisms activated by LiCl in normal tissues are modulated in some cancers. The inhibition by LiCl entails various mechanisms including activation of p53 and NF-κB signaling [12,16] however very little is known about the role of the Wnt pathway in this process. Given the importance of the non-canonical Wnt pathway in constraining the canonical pathway in normal tissues, and the dysregulation of the canonical Wnt pathway in different cancers, the present study was designed to assess the effect of LiCl on human colorectal cancer cell proliferation and investigate the impact of non-canonical Wnt pathway components in these effects. A principal goal of the study was to determine whether non-canonical pathway components regulate β-catenin expression in human colorectal cancer. The results demonstrate that the stimulation of human colorectal cancer cell proliferation *in vitro* can be inhibited by the induction of *Wnt9A* that functions as a non-canonical ligand which results in suppression of β-catenin protein.

## 2. Results

### 2.1. Effect of LiCl on CRC Cell Proliferation and Apoptosis

**Figure 1:**
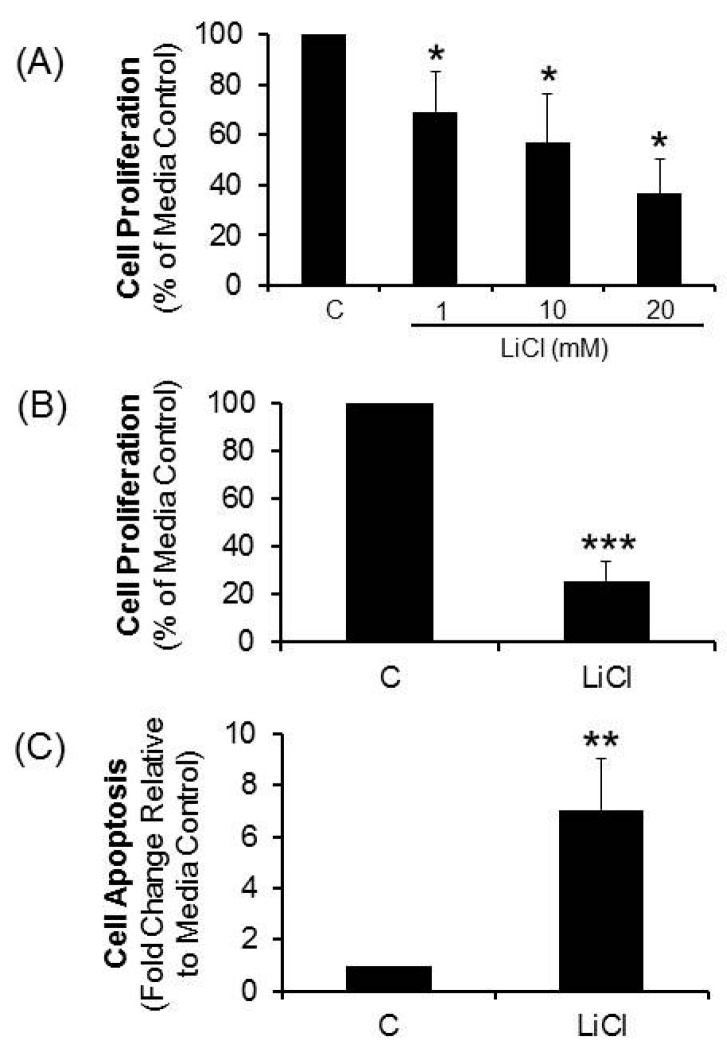
Effect of LiCl on proliferation and apoptosis of colorectal cancer (CRC) cells. CRC cell proliferation and apoptosis was determined in cells treated with LiCl for a period of 72 h. (**A**) Proliferation determined in cells treated with different concentrations of LiCl (*n* = 4); (**B**) Proliferation determined in cells treated with 20 mM LiCl (*n* = 5); (**C**) Apoptosis determined in cells treated with 20 mM LiCl (*n* = 5). Statistical analysis: One way ANOVA with *post hoc* pairwise multiple comparisons using Student-Newman-Keuls method and Student’s *t*-test. * *p* < 0.05; ** *p* < 0.01; *** *p* < 0.001.

Treatment of primary short term colorectal cancer cell lines (*n* = 4) resulted in a concentration-dependent suppression of cell proliferation by LiCl (Figure 1A). At 20 mM, proliferation was inhibited in 5/5 CRC lines (mean ± standard error of the mean (SEM) = 74% ± 18%; *p* < 0.001) relative to media controls (Figure 1B). LiCl elicited significant apoptosis in 5/5 CRC lines (*p* < 0.01, Figure 1C). These results suggest that signaling mechanisms elicited by LiCl that stimulate cell proliferation in normal cells may be dysfunctional in these CRC cells.

### 2.2. Effect of LiCl on β-Catenin Message and Protein in CRC Cells

**Figure 2:**
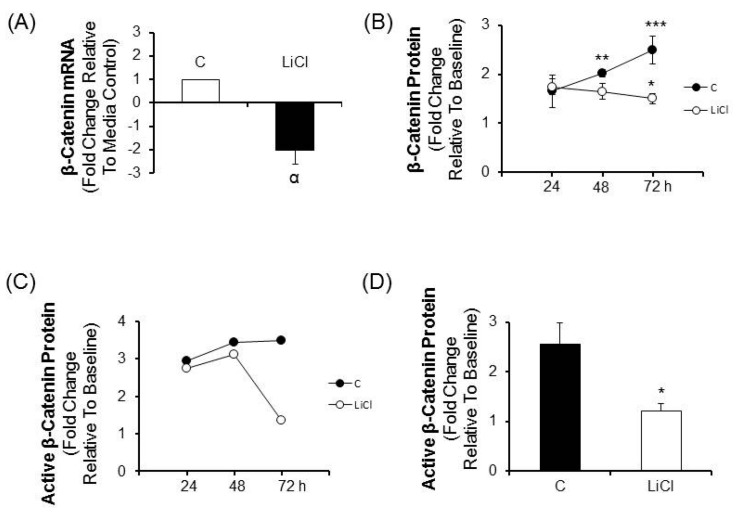
The effect of LiCl on β-catenin message and protein in CRC cells. CRC cells were treated for various periods of times with 20 mM LiCl and β-catenin mRNA and protein were determined. (**A**) β-catenin mRNA in cells treated with LiCl for 72 h (*n* = 4); (**B**) Total β-catenin protein in cells treated for 24, 48 and 72 h (*n* = 5); (**C**) Active β-catenin protein in cells treated with LiCl for 24, 48 and 72 h (*n* = 2); Statistical analysis: (**D**) Active β-catenin protein in cells treated with LiCl for 72 h (*n* = 4). One way ANOVA with *post hoc* pairwise multiple comparisons using Student-Newman-Keuls method and Student’s *t*-test. ^α^ *p* < 0.03; * *p* < 0.025; ** *p* < 0.01; *** *p* < 0.001.

Based on the finding of proliferation inhibition and induction of apoptosis in CRC cells treated with LiCl, studies to assess canonical Wnt pathway activity were performed. Because canonical Wnt signaling requires stabilization of β-catenin leading to stimulation of TCF/LEF mediated transcription, the effects of LiCl treatment on β-catenin mRNA and protein was determined. Relative to media controls, LiCl elicited a 2-fold decrease of β-catenin mRNA (*p* < 0.03; Figure 2A). Total β-catenin levels increased significantly from baseline in media treated cells (*p* < 0.01 and 0.001 at 48 and 72 h respectively). In response to LiCl the total β-catenin levels were significantly decreased relative to media treated cells at 72 h (*p* < 0.025, Figure 2B) and were lower than baseline but not in a statistically significant manner. Comparable results were obtained for measurements of the active form of β-catenin in media and LiCl-treated cells which demonstrated a precipitous drop relative to media-cultured cells by 72 h (Figure 2C,D).

### 2.3. Effect of LiCl on the Expression of Wnt Pathway Components in CRC Cells

**Figure 3:**
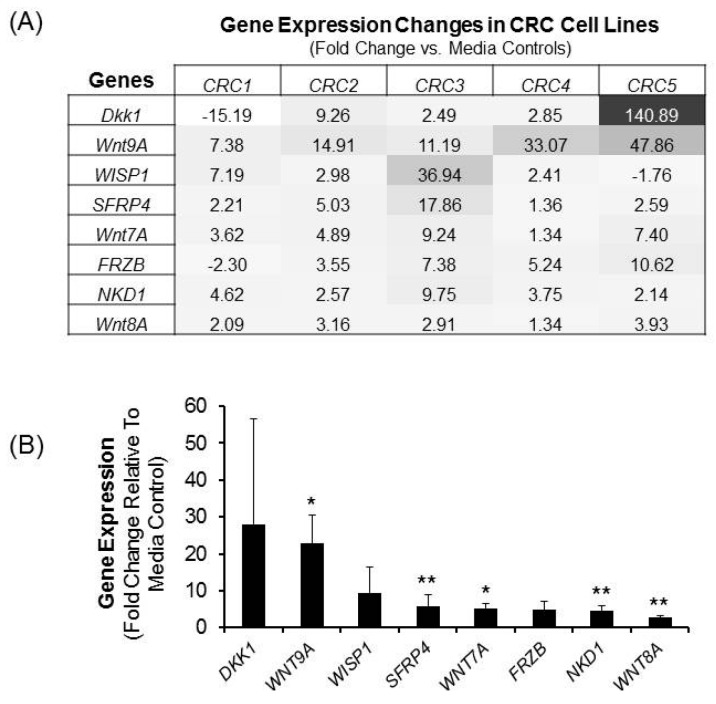
The effect of LiCl on the expression of Wnt signaling components. CRC cells were treated with 20 mM LiCl for a period of 72 h and gene expression analysis was performed. (**A**) Heat map showing the intensity of Wnt pathway gene expression changes induced by LiCl in all five CRC cells. Data shown is of gene expression changes of two-folds or greater in four or all five cell lines; (**B**) Summary of the LiCl-mediated changes in gene expression from the heat map in panel **A**. The data are expressed as means ± SEM. of fold change relative to media control from 5 distinct CRC cell lines. Statistical analysis: Student’s *t*-test. * *p* < 0.025; ** *p* < 0.01; *n* = 5. The intensity of gene expression increases from white to black with the black color representing genes expressed the greatest.

Given the capacity of the non-canonical Wnt pathway to suppress canonical Wnt signaling, studies to assess the effects of LiCl on non-canonical Wnt pathway components were undertaken. Initial exploratory studies relied on evaluating the effects of LiCl on expression of genes related to the Wnt pathway. In Figure 3A, Wnt genes modulated by ≥2 folds are depicted for at least 4 of the 5 cell lines. It can be appreciated that the expression of individual Wnt pathway genes across these 5 cell lines was qualitatively variable (Figure 3A) with the exception of two genes, namely Wnt ligand *Wnt9A* and naked cuticle homolog 1 (*NKD1*). With respect to *Wnt9A*, LiCl increased expression by an average of 23-folds (range = 7–48; *p* < 0.025; Figure 3B) while expression of *NKD1* was increased by an average of 4.5-folds (*p* < 0.01; Figure 3B). *Dkk1* expression was affected substantially by LiCl, but the effects were qualitatively inconsistent ranging from an increase of 141-fold in 1 cell line, to a decline of 15-fold in another. Other genes increased ≥2 folds in some but not all cells lines, which achieved statistical significance by a pooled analysis, included ligands *Wnt8A* (*p* < 0.01); *Wnt7A* (*p* < 0.025); and the Wnt inhibitor *SFRP4* (*p* < 0.01; Figure 3B).

### 2.4. Effect of Wnt Ligand Synthesis/Secretion on LiCl-Mediated Proliferation Inhibition

**Figure 4:**
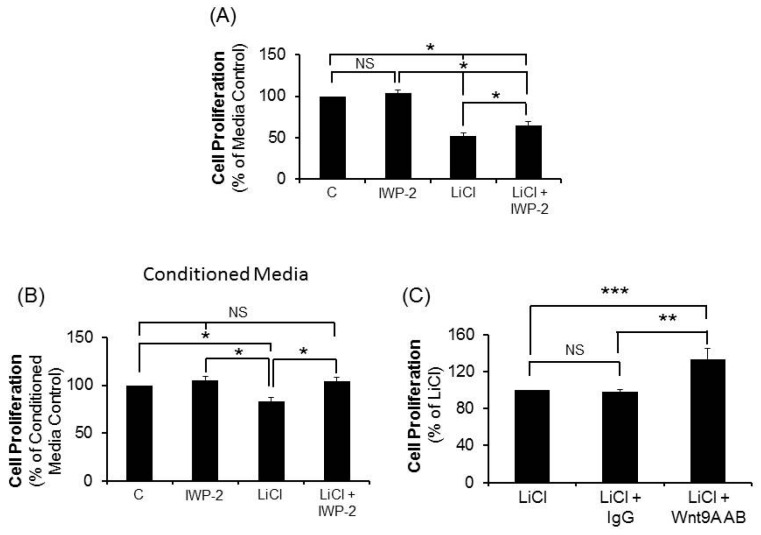
Effect of IWP-2 and *Wnt9A* antibody on LiCl-mediated suppression of CRC cell proliferation. (**A**) CRC cells were treated with 20 mM LiCl with or without 1.0 µM IWP-2 (*n* = 4); (**B**) Effect of conditioned media from cells treated with media, LiCl, IWP-2 or LiCl+IWP-2 on CRC proliferation (*n* = 4); (**C**) Cell proliferation was determined in CRC treated with LiCl, LiCl + IgG, or LiCl + *Wnt9A* antibody (*n* = 5). The data are expressed as means ± SEM. of fold change relative to media or LiCl controls. Statistical analysis: One way ANOVA with *post hoc* pairwise multiple comparisons using Student-Newman-Keuls method. * *p* < 0.05; ** *p* < 0.01; *** *p* < 0.005. NS = not statistically significant.

The gene expression results revealed that several Wnt pathway components that are increased in response to LiCl are ligands. Thus, our next studies investigated the effect of blocking Wnt ligand production on CRC cell proliferation. The production of a Wnt ligand involves several steps including acylation by porcupine, a protein-cysteine *N*-palmitoyltransferase that is inhibited by IWP-2 [17]. We tested various concentrations of IWP-2 on 20 mM LiCl-mediated effects on two CRC cell lines and determined that IWP-2 reversed the LiCl-mediated suppression of CRC cell proliferation in a concentration-dependent manner with 1.0 μM representing the most effective concentration. We therefore utilized 1.0 μM concentration of IWP-2 for our subsequent studies. The results reveal that IWP-2 partially but significantly reversed the suppression of CRC cells by LiCl (*p* < 0.05; Figure 4A) implicating a role for the production Wnt ligands in LiCl-mediated proliferation inhibition. To test this possibility further and to explore if this effect was due to a secreted ligand, culture media was generated with cells treated with LiCl in the presence or absence of IWP-2 and used to treat the matched cell lines for 72 h prior to measurement of proliferation. The results demonstrate that cells treated with conditioned media from the autochthonous LiCl-treated cell line significantly inhibited proliferation (*p* < 0.05, Figure 4B) relative to conditioned media from untreated cells. Furthermore, inhibition was absent in cells treated with conditioned media from LiCl-treated cells that were co-incubated with IWP-2 (Figure 4B).

The studies investigating Wnt pathway gene expression, ligand synthesis/secretion, and the relationship to LiCl-mediated proliferation inhibition of human CR cells are consistent with a role for a non-canonical pathway ligand in these effects. As noted above, the most consistent effect of LiCl with respect to gene expression, both qualitatively and quantitatively across all five cells lines were on the gene for the non-canonical ligand, *Wnt9A*. Thus, our next experiments investigated the effect of specific *Wnt9A* antibody on LiCl-mediated CRC cell proliferation inhibition. The results show that LiCl-mediated inhibition of CRC cell proliferation was reversed significantly in all cell lines by the specific *Wnt9A* antibody (*p* < 0.005; Figure 4C), whereas there was no effect elicited by an isotype-matched, control IgG.

### 2.5. Effect of Recombinant Wnt9A Protein on Proliferation, Apoptosis and Active β-Catenin Protein Levels in CRC

**Figure 5:**
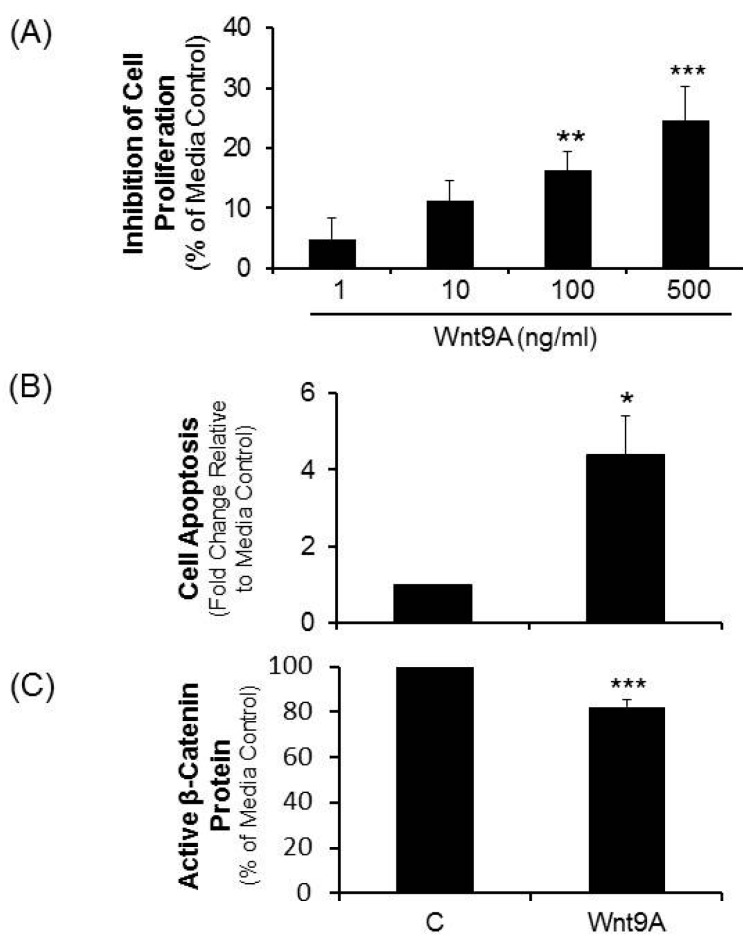
Effect of recombinant *Wnt9A* protein on CRC cell proliferation, apoptosis and active β-catenin levels. CRC cells were treated with recombinant human *Wnt9A* protein for a period of 72 h. (**A**) Proliferation determined in cells treated with a range of *Wnt9A* protein; (**B**) Apoptosis was determined in cells treated with 500 ng/mL *Wnt9A* protein; (**C**) Active β-catenin protein in cells treated with 500 ng/mL *Wnt9A* protein. Statistical analysis: Student’s *t*-test. * *p* < 0.03; ** *p* < 0.01; *** *p* < 0.001; *n* = 5.

The collective results implicating a role for the non-canonical ligand, Wnt9A in mediating suppression of proliferation in human CRC cells treated with LiCl was evaluated further using recombinant Wnt9A protein. Dose titration studies demonstrated that recombinant Wnt9A protein suppressed the proliferation of CRC proliferation in a concentration dependent manner which, at 500 ng/mL, led to proliferation inhibition of 24% (*p* < 0.001, Figure 5A). Moreover, at this concentration Wnt9A was shown to increase apoptosis (*p* < 0.03, Figure 5B), as well as suppress active β-catenin protein levels (*p* < 0.001, Figure 5C).

### 2.6. Schematic Representation of the Mechanism Involved in LiCl-Mediated Suppression of CRC Cell Proliferation

**Figure 6:**
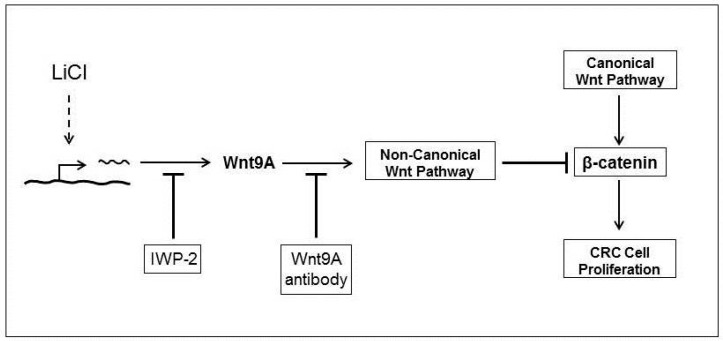
Mechanism for the LiCl-mediated suppression of CRC cell proliferation. Schematic shows the mechanism involved in the LiCl-mediated suppression of CRC cell proliferation. Blunted arrows indicate inhibition. Dotted line indicates unknown mechanism.

The mechanism utilized by LiCl for the suppression of CRC cell proliferation is depicted in Figure 6. It shows that the suppression of CRC cell proliferation is in part due to induction and *Wnt9A* that regulates β-catenin activity. Suppression of *Wnt9A* induction by IWP-2 or sequestering of the ligand using antibody results in reversal of LiCl-mediated effects.

## 3. Discussion

Given the widely recognized action of LiCl as an activator of β-catenin through the canonical Wnt pathway resulting in stimulated cellular proliferation [14], in contrast to our demonstration of LiCl-mediated proliferation inhibition of human CRC cells from surgical specimens, the current investigation was designed to characterize LiCl’s effect(s) on the Wnt pathway in human colorectal cancer. The results demonstrate that LiCl suppresses CRC cell proliferation by a mechanism that entails increased expression and secretion of a non-canonical Wnt ligand, Wnt9A in conjunction with suppression of β-catenin protein levels. Thus, LiCl-mediated inhibition of both cellular proliferation and active β-catenin protein levels were replicated by recombinant *Wnt9A* protein linking this non-canonical Wnt ligand to the effects of LiCl. Similarly, the reversal of these effects in LiCl-treated CRC cells by non-specific Wnt ligand synthesis/secretion inhibitor, and by specific inhibition of Wnt9A with specific antibody, further supports the contention that the activity of the canonical Wnt pathway in human CRC can be attenuated by increasing the expression of some components of the non-canonical Wnt pathway. That well tolerated agents such as LiCl have the capacity to do this *in vitro* suggests that a similar approach *in vivo* may be therapeutically beneficial.

A principal feature of aberrant Wnt signaling characteristic of malignancy is hyperactivation of the canonical pathway in association with dysregulation of β-catenin. One consequence of this aberrant regulation is attenuated degradation of β-catenin resulting in chronic activation of genes that promote cell proliferation. However, recent studies indicate that the elevation and persistence of β-catenin levels alone are insufficient to explain the abnormal proliferative behavior of cancer cells and that additional modulators are required for the development of the neoplastic phenotype [18,19]. Given the importance of the non-canonical pathway in constraining the ability of the canonical pathway to promote cell proliferation, it is conceivable that disturbances in induction and/or function of various non-canonical pathway components are involved in neoplasia. This hypothesis is supported by studies showing that non-canonical pathway genes are suppressed in cancer tissues [8,9,10].

Nevertheless, how suppression of non-canonical pathway genes is related to neoplasia is not completely understood. A plausible explanation worth further investigation is that constraints imposed by the non-canonical pathway in normal tissues are absent in cancer cells due to inhibition of non-canonical pathway components. This idea is supported by studies which show that forced expression of non-canonical components in tumor cell lines results in decreased β-catenin signaling concomitant with decreased colony formation and cellular proliferation [10].

*Wnt9A*, formerly known as *Wnt14*, has been classified as both a canonical [20,21] and a non-canonical ligand [22,23,24]. The type of signal that is activated depends on the milieu of signaling components that are expressed in cells [24,25]. Ligands that stabilize β-catenin protein leading to increased cytosolic levels which can then translocate to the nucleus and bind to TCF/LEF transcription factors to activate genes that stimulate proliferation are considered canonical. Ligands that counter mechanisms that stabilize β-catenin protein levels are considered non-canonical. Based on the observations herein, and the studies cited, we conclude that *Wnt9A* functions as a non-canonical ligand in human CRC cells.

The substantial and consistent upregulation of *Wnt9A* gene expression in LiCl-treated CRC cells (ranging from 7- to 48-fold across 5 short term lines) that we observed in association with proliferation inhibition (ranging from 58% to 98%), coupled with the literature cited here, justified investigating the contribution of Wnt9A in these effects. In addition, the results obtained by: blocking ligand secretion; treatment with specific anti-Wnt9A antibody; and treatment with recombinant Wnt9A protein all point to a role for *Wnt9A* in the suppression of CRC cells we report herein. The suppression of β-catenin protein levels by Wnt9A which emulate LiCl effects further adds to this notion and indicates that the suppression of CRC cell proliferation is mediated in part through the activation of the non-canonical pathway that inhibits the canonical pathway. Nevertheless, while all these maneuvers produced results that were quantitatively significant and qualitatively consistent with a role for Wnt9A as a tumor suppressor, the magnitude of proliferation inhibition observed cannot be attributed to the effects of Wnt9A alone. Thus, other genes or factors must be involved.

One such possibility is the *NKD1* gene. In conjunction with *Wnt9A*, *NKD1* was the only other gene that was significantly upregulated in all cell lines. *NKD1* is a protein that functions as a negative feedback inhibitor of the Wnt pathway but has the capacity to modulate both canonical and non-canonical signaling pathways [26]. It is, therefore, conceivable that this gene may act in concert with *Wnt9A* to enhance the suppressive effects of *Wnt9A* observed with LiCl. Further studies are needed to determine this possibility.

The contribution of Wnt pathway proteins that were significantly upregulated in response to LiCl in at least some of the 5 cell lines used in this study may also merit further investigation. Additional candidates based on our studies include: *Dkk1*, *SFRP4*, *Wnt7A* and *Wnt8A*. *Dkk1* is a potent inhibitor of the canonical Wnt pathway as well as a target. The activation of the canonical pathway increases its expression which results in feedback inhibition of the pathway [27]. This is also true for *SFRP4* which can bind to Wnt ligands and inhibit both pathways [2]. The Wnt ligands *Wnt7A* and *Wnt8A* are considered non-canonical and canonical ligands, respectively [28]. These components were not investigated in this study due to the inconsistent effects of LiCl, qualitatively and quantitatively on their genes when considering all 5 cell lines. Nevertheless, the partial reversal of the effects of LiCl in these lines by *Wnt9A* strongly suggests that further studies assessing the contribution of other non-canonical Wnt pathway components are justified.

The results presented herein suggest that the non-canonical Wnt ligand, *Wnt9A* has the potential to act as a tumor suppressor in cells that have sustained oncogenic mutations sufficient to hyperactivate β-catenin action. Studies that demonstrate decreased expression of the *Wnt9A* gene in different cancers including colorectal cancer are consistent with this possibility [29]. Such a role is also inferred by the demonstration that increased *Wnt9A* expression is related to apoptosis and cell cycle arrest [30] as well as by studies which show that knocking down expression of *Wnt9A* leads to increased proliferation of breast cancer cells [23]. In colorectal cancer, frameshift mutations in the *Wnt9A* gene sufficient to result in loss of function of the protein have been reported [31].

The principal objective for this study was to investigate the effect of various components of the non-canonical Wnt pathway in the LiCl-mediated suppression of colorectal cancer cell proliferation and to determine if such effects involve regulation of β-catenin expression. That objective was met with the discovery that the expression of a non-canonical Wnt ligand, *Wnt9A* was significantly increased by LiCl and that this ligand inhibited colorectal cancer cell proliferation in association with suppression of β-catenin expression (Figure 6). These observations justify additional studies to fully comprehend the mechanism that leads to the suppression of colorectal cancer cells by LiCl. For example, the elucidation of the mechanism for the induction of the non-canonical Wnt ligand would be of vital importance since the mechanism for the expression of Wnt ligands, in general, is not fully understood. Such an understanding could lead to the development of therapeutic interventions that stimulate induction of non-canonical Wnt ligands that counteract the effects of the proliferative canonical pathway. It is conceivable that studying the effects of LiCl on GSK-3β could potentially lead to the elucidation of the mechanism utilized for the induction of the Wnt ligands.

In conclusion, this is the first study to demonstrate induction of a non-canonical ligand, *Wnt9A* as part of a mechanism for LiCl-mediated suppression of CRC proliferation. The LiCl-mediated suppression is related to the activation of the non-canonical pathway but the involvement of other pathways is plausible. Nevertheless, the results of this study also suggest that one consequence of malignant transformation may be the suppression of non-canonical Wnt pathway activity, or the release of canonical Wnt pathway control by non-canonical pathway components. In human CRC, our results favor the first possibility by demonstrating that stimulating the non-canonical pathway restores canonical pathway regulation. Thus, agents that upregulate the non-canonical pathway components, especially *Wnt9A*, and activate this pathway may be therapeutically beneficial in human colorectal cancer.

## 4. Materials and Methods

### 4.1. Study Design

Short term, primary cell lines were established from resected colorectal tumor specimens, classified and characterized by the attending surgical pathologist on each case. The tumors were obtained under an institutional review board approved protocol, from five patients (age range = 40–64 years) who were undergoing treatment for colorectal adenocarcinoma. Short term, primary CRC cell lines (*n* = 5) were established from resected tumors from patients with metastatic and/or recurrent disease. CRC were treated with LiCl (Sigma-Aldrich Corp., St. Louis, MO, USA), an activator of the canonical Wnt pathway (CWP) in the presence and absence of various Wnt pathway modulators including: IWP-2 (Sigma-Aldrich Corp.), a pan inhibitor of CWP and NCWP Wnt ligand secretion; conditioned media (CM) from LiCl ± IWP-2 treated cells; a specific antibody against Wnt ligand, *Wnt9A* (Santa Cruz Biotechnologies, Inc., Santa Cruz, CA, USA); and recombinant *Wnt9A* protein (Genemed Biotechnologies, Inc., San Francisco, CA, USA). Cell proliferation and apoptosis assays and RNA isolation for quantitative PCR gene expression array analysis were performed at 72 h. ELISA was performed on cell lysates at 24, 48 and 72 h.

### 4.2. Tumor Tissues Procurement from Patients

The protocol entitled “The BioSample/Tissue Repository at Midwestern Regional Medical Center, Inc.” was approved by the Western Institutional Review Board, Panel #17. As per the IRB protocol, the specific requirement to access the tumors required patient’s written informed consent for the tumor repository operations which include: (1) storage of the extra tissues not needed for clinical care; and (2) permission to conduct research on these tissues stipulating that all identifiers are stripped from the specimen and can only be matched to the patient and their clinical data through a code provided by the repository. The code is under the control of the principal investigator, Tan, Chair of the Department of Pathology and Laboratory Medicine, and not shared with any of the investigators who use the tissues in the repository.

### 4.3. Cell Culture

Tumor lines were passaged in RPMI 1640 medium supplemented with 10% heat-inactivated fetal bovine serum (Corning, Manassas, VA, USA) and 50 μg/mL Gentamicin (Sigma-Aldrich Corp., St. Louis, MO, USA). Tumor specimens received from surgery were dissected into 1 mm slices and subjected to a collagenase/DNase (0.14%/0.1%, Sigma) digestion at 37 °C for 1–2 h. After digestion, specimens were strained, washed in HBSS (Sigma), and put into culture.

### 4.4. Wnt Pathway Focused Gene Expression Arrays

Transcriptional expression of genes was determined on the extracted RNA using 96-well real-time PCR arrays. cDNA was synthesized using the RT^2^ First Strand cDNA Kit (SABiosciences Corp., Frederick, MD, USA) according to the manufacturer’s instructions. Transcriptional gene expression was performed using the Human Wnt Signaling Pathway Plus RT^2^ Profiler PCR Array System (SABiosciences Corp., Frederick, MD, USA), according to the manufacturer’s instructions. Real-time PCR was performed using the MyiQ Real-time PCR detection system (Bio-Rad Laboratories Inc., Hercules, CA, USA). All transcriptional gene expression analyses were performed on the SABiosciences Corp. web portal using the RT^2^ Profiler PCR Array Data Analysis program (version 3.2). Transcriptional gene expression was defined as fold-change *versus* media controls.

### 4.5. ELISA for Total and Active β-Catenin

Total and active β-catenin levels were determined by ELISA by the Human Total β-Catenin DuoSet IC (R&D Systems, Minneapolis, MN, USA). To determine the active β-catenin levels, the capture antibody provided with the kit was replaced by Anti**-**Active**-**β**-**Catenin (anti**-**ABC), clone 8E7 (EMD Millipore, Billerica, MA, USA) [32]. Cells were subjected to various treatments for 24, 48 or 72 h and lysed by the addition of the lysis buffer. ELISA was performed on cell lysates according to the manufacturer’s instructions.

### 4.6. Cell Proliferation Assay

Cell proliferation was measured by a standard MTS Assay (Promega, Madison, WI, USA). A total of 5000 cells in logarithmic growth phase were inoculated into treatment wells in quadruplicate. Cells were subjected to various treatments (indicated above) and incubated for 72 h. Three hours before the end of the incubation period, MTS reagent was added to the plates and absorbance readings were performed on a multimode plate reader (Enspire 2300-001L, PerkinElmer, Waltham, MA, USA) at 490 nm.

### 4.7. Apoptosis Assay

Cell apoptosis was determined using Caspase-3 Colorimetric Assay kit (R&D Systems, Minneapolis, MN, USA). Cells were subjected to various treatments for 72 h and lysed by the addition of the lysis buffer provided with the kit. The Caspase-3 assay was performed on cell lysates according to the manufacturer’s instructions using a multimode plate reader (Enspire 2300-001L, PerkinElmer, Waltham, MA, USA). The results are expressed as fold change relative to media controls. 

### 4.8. Statistical Analysis

The data were expressed as means ± standard error of the means (S.E.M). Student’s *t*-test and One-way analysis of variance (ANOVA) with *post hoc* pairwise multiple comparisons using the Student-Newman-Keuls method was performed to determine significance of differences between treatments. *p*-Values of less than 0.05 were considered significantly different.

## 5. Conclusions

This is the first study to demonstrate induction of a non-canonical ligand, *Wnt9A* as part of a mechanism for LiCl-mediated suppression of CRC proliferation. Inducing components of the non-canonical Wnt pathway may be therapeutically beneficial for attenuating uncontrolled proliferative canonical signaling in malignant tissues.
